# A Robust Linear Feature-Based Procedure for Automated Registration of Point Clouds

**DOI:** 10.3390/s150101435

**Published:** 2015-01-14

**Authors:** Martyna Poreba, François Goulette

**Affiliations:** MINES ParisTech, PSL–Research University, CAOR–Centre for robotics, 60 bd St Michel, 75006 Paris, France; E-Mail: francois.goulette@mines-paristech.fr

**Keywords:** matching, alignment, transformation, registration, point cloud, feature, line, quality, distance

## Abstract

With the variety of measurement techniques available on the market today, fusing multi-source complementary information into one dataset is a matter of great interest. Target-based, point-based and feature-based methods are some of the approaches used to place data in a common reference frame by estimating its corresponding transformation parameters. This paper proposes a new linear feature-based method to perform accurate registration of point clouds, either in 2D or 3D. A two-step fast algorithm called Robust Line Matching and Registration (RLMR), which combines coarse and fine registration, was developed. The initial estimate is found from a triplet of conjugate line pairs, selected by a RANSAC algorithm. Then, this transformation is refined using an iterative optimization algorithm. Conjugates of linear features are identified with respect to a similarity metric representing a line-to-line distance. The efficiency and robustness to noise of the proposed method are evaluated and discussed. The algorithm is valid and ensures valuable results when pre-aligned point clouds with the same scale are used. The studies show that the matching accuracy is at least 99.5%. The transformation parameters are also estimated correctly. The error in rotation is better than 2.8% full scale, while the translation error is less than 12.7%.

## 1. Introduction

Over the last decade, laser scanning systems have proven to be an efficient measurement tool providing satisfactory accuracy in a variety of applications, notably in 3D reconstruction and realistic modeling of the environment. However, a technique is needed to register and visualize the resulting 3D scans in a common coordinate system. The aim is to estimate transformation parameters, which minimize a chosen measure of mismatch between overlapping point clouds. With static terrestrial laser scanning (TLS), an object is collected from different scanner locations in order to avoid the presence of so-called dead spots and to recover a dense 3D point cloud spanning its entire surface. As a consequence, the alignment phase faces the issue of merging multi-view scans together. In addition, a registration problem arises if the same area is acquired more than once. In the case of airborne (ALS) or ground-based (MLS) mobile scanning platforms, offsets between overlapping areas of adjacent flying strips or surveys resulting from drifts of the INS or temporary loss of GNSS signals may occur. Finally, the wide variety of available measurement techniques provides different sources of 3D information requiring integration into one coherent dataset.

Both rigid and non-rigid alignment methods exist. The classification of registration approaches in [1] divides algorithms into three main families. The first are multi-feature-based methods relying on the extraction of primitives, which achieve the alignment through prior correspondence of geometric features. As such, these algorithms depend on the existence of suitable primitives within the scene. Moreover, the data filtering and segmentation steps can take considerable amounts of processing time. A second solution consists of rendering 3D point clouds into a surface model (e.g., meshing). The third and final category are point-based methods, which require neither feature recognition nor pre-modeling. The most commonly employed solution is the well-known Iterative Closest Point (ICP) algorithm [2], a robust and accurate method provided that a good rough alignment exists. The ICP iteratively minimizes the point-to-point distances between overlapping point clouds by estimating transformation parameters, then re-pairing points in the transformed clouds. These two main steps are repeated until the registration error is smaller than a given threshold value. Despite its numerous advantages, the ICP method is burdened by limitations, which are still under study. Many variants have been proposed to improve the individual steps of the ICP namely the selection of points, matching, weighting and rejection of erroneous associations, and choice of cost function to be minimized [3–5]. Nonetheless, the main weaknesses of the ICP algorithm are the high number of iterations needed to achieve convergence, the requirement for a good initialization, and sensitivity to noise. Furthermore, the ICP's matching step is the most time-consuming part of the registration phase [1], and the initial alignment is usually assigned by hand. A wide variety of research has focused on automating the coarse registration and reduction of matching time. A current trend is two-step approach [6,7]. At first, a feature-based coarse registration provides an initial estimate, which is then passed to an ICP-like method for accurate and robust fine registration. Referring to [8], the ICP algorithm is still only well-formulated for pair-wise registration (*i.e.*, two scans at the time). Therefore, other methods are needed to ensure simultaneous alignment of multiple point clouds.

In summary and as stated in [9], the registration process should address four issues: (1) Extracting corresponding geometric primitives within the scans, such as key-points, linear, planar, spherical or cylindrical features as well as higher-level shape descriptors; (2) Estimating the transformation parameters that ensure the best fit; (3) Defining the similarity measure for conjugate features, which describes their coincidence after the registration process; (4) Choosing the matching strategy. In this context, a solution to each of the above-mentioned tasks is proposed in this paper.

### 1.1. Motivation

Man-made urban areas are rich in linear features, making their use appropriate in several applications. They can be also useful to carry out automatic 2D or 3D registration between different scans as well as for integration of LiDAR and photogrammetric data. From an algorithmic point of view, the determination of 3D motion from line correspondences is more challenging compared to point-based methods. However, the linear primitives, recovered from plane-plane intersections, are easier to extract and describe, even within low-density and relatively noisy point clouds. Rather than using conjugate points, linear features are thus employed in this research. Hence, to obtain the rigid-body transformation parameters, at least two non-parallel lines are required (since a single line has three degrees of freedom). The pair-wise registration of datasets that are already geo-referenced and do not exhibit large changes, especially in rotation is thus focused. Indeed, a good *a priori* alignment between the point clouds should be assumed, meaning that it needs to be only refined by the co-registration. The objective is to develop an effective registration procedure for the co-alignment of the Mobile Laser Scanning (MLS) point cloud data. Therefore, a complete framework for the so-called Robust Line Matching and Registration (RLMR) is outlined.

The remainder of the paper is organized as follows: after a short presentation of the existing optimization methods suitable to estimate the transformation parameters from line segment correspondences, primitives, which can be reliably extracted from the MLS data, are introduced. In Section 2, a procedure to precisely register, even in noisy data conditions, two point clouds is described. The performance and feasibility of the proposed methodology are evaluated through experimental results using both simulated and experimental datasets (Section 4). Finally, some conclusions and recommendations for future work are drawn (Section 5).

### 1.2. Overview of Linear Feature-Based Registration Methods

A variety of methods have been proposed to tackle the problem of estimation of transformation parameters from pre-matched lines [9–18]. Among these various algorithms [10] proposes two variants of the so-called Iterative Closest Line (ICL) algorithm. The first one, the ICP form, has the same logic as the ICP algorithm provided that one considers only the estimation of rotation (closed-form solution). However, the translation computation is performed differently. The second variant, the ICL (alternative form), is inspired by [11] and seeks to determine seven parameters (rotations, translations, and scale factor) of a 3D Helmert transformation. In our case, the scale factor is considered unity since the laser rangefinders provide the true scale. Nevertheless, this latter approach requires that matched elements have the same length and their endpoints are conjugate to each other. To overcome limitations of such explicit assumption [10], suggests matching the length of corresponding linear features before starting the algorithm. Another improvement of algorithm [11] is given by [12]: an additional unknown vector defined for corresponding lines compensates the non-correspondence of endpoints, then, a point-based approach using a weight-modification process is implemented. In this way, the systematic error resulting from the use of non-conjugate points is eliminated. These two optimization methods are nonlinear and require prior information about the unknown transformation parameters. When the initial estimate is sufficiently distant from the true one, this method may provide an incorrect solution. A qualitative and quantitative assessment was carried out by [13] to analyze the performance of the method proposed in [12]. The transformation parameters are compared with those obtained from other methods, *i.e.*, the Iterative Closest Projected Point (ICPP) [14] (a variant of the ICP approach) and a planar feature-based registration method [19]. On the basis of this study [9] developed a two-step process where the coarse alignment between overlapping lines is refined through an ICPP registration.

Using different representations of line segments and rotation, [15] analyzed several methods for the estimation of transformation parameters e.g., the Extended Kalman Filter (EKF), the Singular Value Decomposition (SVD) and a general minimization process. They observed that the application of a general minimization algorithm is very time-consuming compared to SVD. The EKF with the rotation-axis representation was found to be the preferred way to achieve a high-accuracy solution. In turn, the quaternion representation of rotation combined with the SVD (the so-called EIGEN method) is efficient and does not need an initial estimate. However, the results are not very representative.

Meanwhile [16], suggest the use of randomly selected line triplets from all possible permutations. The objective is to test a very large number of transformations and to keep only the best one. However there is an implicit assumption that the midpoints of the corresponding line segments are conjugates of each other, which provides the correct results only in particular situations.

Finally [17], develop a four-variant algorithm (iterative optimization algorithm) for estimating an optimal transformation giving the best fit of an image set being moved (I) to a model reference set (M). The (closed-form) solution for equal-length line segments is described in [18]. In the case of different lengths, three approaches are possible: (1) Finite Model, Finite Image (FMFI)—both sets consist of line segments; (2) Infinite Model, Infinite Image (IMII)—both sets contain infinite lines; (3) Finite Model, Infinite Image (FMII)–mixed case. Regardless of the choice, a method that yields the transformation parameters without need for an initial approximation is proposed. The cost function is formulated explicitly in terms of corresponding points on each pair of homologous lines. These corresponding points need to be determined iteratively. In summary, irrespective of the method chosen, the estimation process addresses two separate tasks: a non-linear stage to recover the rotation and a linear stage to obtain the translation.

## 2. Methodology

**Figure 1.**
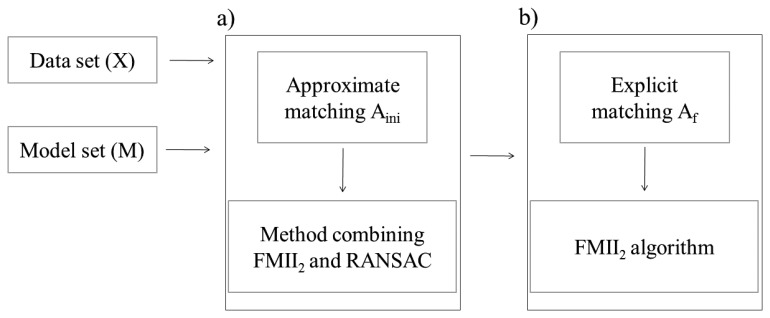
RLMR procedure (**a**) Rough alignment; (**b**) Fine alignment.

The proposed two-stage procedure RLMR consisting of a rough followed by a fine alignment phases handles the registration (Figure 1). Assuming that there is no scale difference between the two overlapping point clouds, the transformation from the Data set (X) to the Model set (M) requires the estimation of three rotation angles and three translations. For this task one could implement one of the approaches from the state-of-the-art. The correspondences between two sets of lines are created twice with the algorithm described below. This correspondence-finding algorithm is an extension of the work presented in [20] by providing more details and presenting new results. An analysis of a line-to-line distance (score) leads to identification of conjugate linear features between two sets. This score is composed of angular deviation and spatial separation values. First, a one-to-one correspondence A_ini_ (approximate matching), which is used to find the initial guesses of the unknown transformation parameters should be made. Then, all newly matched line segments (within an explicit matching A_f_ which allows both one-to-many and one-to-null relations between lines) are used to refine the transformation.

Using the incorporated registration primitives, an iterative robust approach is proposed in [17] to eliminate the need for an initial estimate during the estimation process. Knowing that the FMFI algorithm may fail if in a pair of lines the shorter segment is not fully contained within the longer one, the FMII_2_ approach is implemented. In this case, linear features in the Data set are infinite while those in the Model set are finite. Thus, the FMII algorithm has been slightly modified in order to estimate the rigid transformation. The FMII_2_ name is used to distinguish the procedure from its original variant. The transformation parameters for both registration stages are obtained from the FMII_2_ algorithm. To overcome the problem that A_ini_ may contain erroneous pairs resulting from the distance between Data and Model, only three pairs of matched lines selected from A_ini_ are considered when computing the initial transformation. The coarse registration is carried out using an efficient RANSAC algorithm coupled with FMII_2_ in order to provide the best available triplet of pairs within A_ini_. The resulting roughly-aligned datasets permit an explicitly matching A_f_ due to their mutual displacement being substantially reduced. The rough alignment step is paramount because the correspondence-finding procedure is sensitive to distance, or conversely having the two sets close improves matching.

### 2.1. Finding Features of Interest

Linear features reconstructed from 3D point clouds may be divided into two types [10]: (1) crease edges corresponding to discontinuities in the first derivative or equivalently in the surface normal direction, which can be found from plane-plane intersections; (2) jump edges related to discontinuities of the surface.

**Figure 2.**
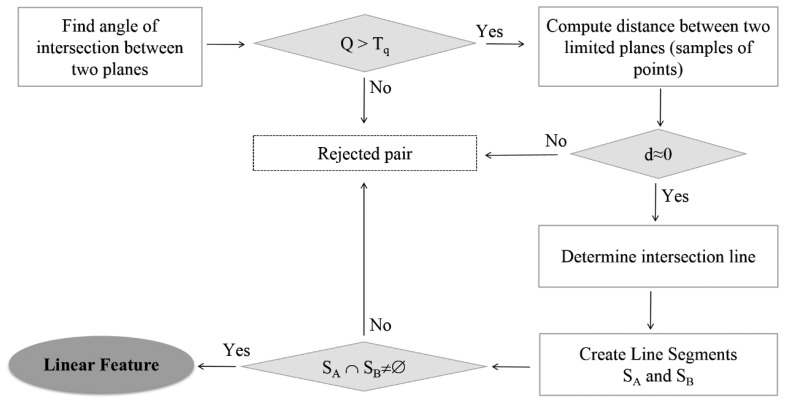
Overview of the plane-plane intersection recovery algorithm.

To minimize problems with recognition and identification of features, only edges formed by intersections between principal planes (typically walls and roads) are considered. This choice of geometric primitives ensures their accurate extraction even from low-density, noisy point clouds [21]. The RANSAC algorithm [22], which considers the largest connected component, is employed for the plane segmentation of point clouds. The adjacency relations between points of each detected planar feature are analyzed using an approach taken from graph theory. Once all “big” planes in the scene are found, one proceeds to generate 3D line segments by finding plane-plane intersections. This task can be easily solved using a brute-force approach of searching for adjacent planes among all possible pair-wise combinations. The consecutive rejections of hypothetical intersecting planes are illustrated in Figure 2.

The concept is straightforward and consists of analyzing the spatial relationships between planes. Let us consider two planes Π_A_ and Π_B_, each one characterized by a polynomial model (using the point-normal form of the equation) fitted to a sample of points associated to a given planar surface. Firstly, the weight Q = sin^2^α, where α is the intersection angle, is assigned for all possible combinations of plane pairs in order to discriminate the quality of each possible edge. Obviously, the intersection is defined most accurately if the paired planes are orthogonal (Q = 1). A pair is thus omitted if the weight Q is less than the threshold T_q_ = 0.5. The next step is to check the distance between admissible pairs of planes and retain only adjacent ones. Then, the line of intersection is found and the range of coupled planes can be considered. At all times, there are two line segments S_A_ and S_B_ resulting from the projection of two sets of points (approximated by the best-fitted planes Π_A_ and Π_B_) onto their line of intersection. Only these pairs of planes are retained for which both line segments overlap with each other. In fact, the final linear feature is a common part of two congruent line segments (S_A_ ∩ S_B_). At this stage a minimum length constraint is also introduced, since short lines are not desirable in the registration process because their descriptive attributes are less accurate.

### 2.2. Line Matching

**Figure 3.**
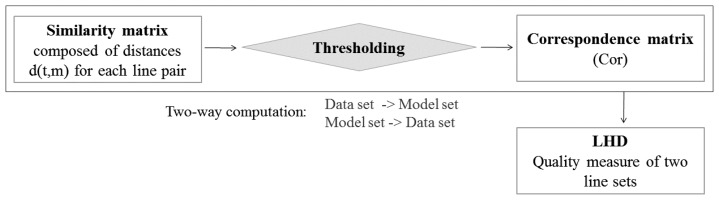
Matching of linear features with quality measure determination.

The determination of correspondences is typically a challenging aspect of the overall registration process, which directly affects the quality of the estimated transformation parameters and has the potential to cause divergence of the optimization algorithm. To explain the conceptual basis of our correspondence-finding procedure, suppose the set Data = {t_1_,t_2_,…,t_i_,…,t_p_} need to be transformed so as to obtain the best alignment with the other set Model = {m_1_,m_2_,…,m_j_,…,m_q_}. Formally, a set is denoted by an uppercase letter and an individual element by a lowercase letter. The identification of conjugate lines involves assigning a dissimilarity measure d(t,m) based on geometric characteristics such as magnitude, orientation and location. This individual score, calculated for the total number of line pair combinations, is used to build a similarity matrix. Afterwards, the same line segments should be identified by analyzing the elements of this matrix. Here “same” means that they have small d(t,m) values (within a predefined threshold). This threshold is fixed and depends on the noise level, the spacing between the two sets, and the accuracy of the line extraction algorithm. All the chosen line pairs to create a binary correspondence matrix are put together. Its indices denote the features, which are most likely to correspond amongst the two input linear feature sets. The three main stages of this method: the similarity matrix, the correspondence matrix formed by thresholding, and the similarity measure Line Hausdorff Distance (LHD) [23] used as a quality indicator, which evaluates the coincidence of conjugate features following the registration procedure, are presented in Figure 3. At all times, the pairing is made bilaterally: the Data set is compared to the Model set and vice-versa. This solution is directed by the conditions imposed by the definition of the Hausdorff metric. This will be further explained in Section 2.2.3.

#### 2.2.1. Similarity Matrix

The main step of our correspondence-finding algorithm is to build a similarity matrix of dimensions *p* by *q* resulting from the cardinality of the Data and Model sets. Thus, the total number of possible matching line pairs will equal the number of lines in the first set (Data) multiplied by the size of second set (Model). Note that in this similarity matrix every column corresponds to a line from the Model set and every row corresponds to a line from the Data set. Each entry of the matrix, referred to as the score d(t,m) for a possible pairing of lines, is calculated according to Equation (1). This metric is inspired by the approach of [23] used to compare two sets of 2D edges, and adapted in [20] to assess the quality of 3D point clouds by means of linear features:

(1) $$\text{d}\left({\text{t}}_{\text{i}},{\text{m}}_{\text{j}}\right)=\sqrt{\text{W}\cdot {\left({\text{d}}^{\alpha }\left({\text{t}}_{\text{i}},{\text{m}}_{\text{j}}\right)\right)}^{2}+{\left({\text{d}}^{\text{II}}\left({\text{t}}_{\text{i}},{\text{m}}_{\text{j}}\right)\right)}^{2}+{\left({\text{d}}^{⊥}\left({\text{t}}_{\text{i}},{\text{m}}_{\text{j}}\right)\right)}^{2}}$$

**Figure 4.**
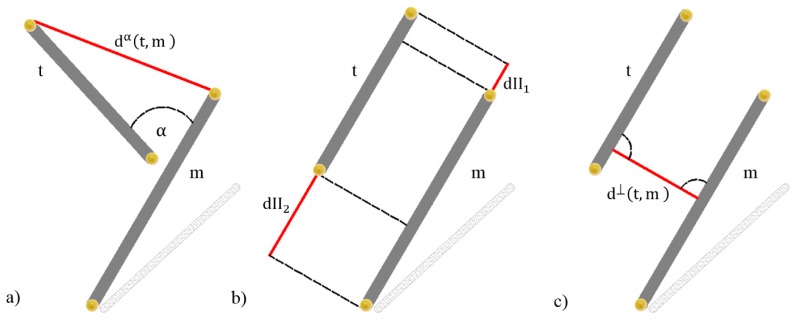
Line displacement measures between two line segments in 3D: (**a**) Angular deviation; (**b**) Overlapping indicator; (**c**) Perpendicular distance.

The angular deviation d^α^(t,m) and the two spatial displacements in the form of an overlapping indicator d^II^(t,m) and a perpendicular distance d^⊥^(t,m) are considered to measure the similarity d(t,m) between any two line segments. The constant W is a non-dimensional weight for angle distance, chosen empirically as 10. Its purpose is to provide a better tolerance to potential misalignments of lines since d^α^(t,m) closes to zero for parallel lines, will much more contribute to the overall value of d(t,m) in case of lack of parallelism. Therefore, the score d(t,m) may be interpreted as a minimum displacement required to superimpose any pair of segments (e.g., t and m). All types of distance defined to measure the dissimilarity between 3D line pairs are shown in Figure 4.

The angular distance d^α^(t,m) is equal to the sine of the intersection angle α between two line directions multiplied by the length of the shorter line segment:

(2) $${\text{d}}^{\alpha }\left(\text{t},\text{m}\right)=\min\left({\text{L}}_{\text{t}},{\text{L}}_{\text{m}}\right)\cdot \sin\alpha$$

Due to the fact that none of the pairs of lines (t_i_,m_j_) are exactly parallel, every Data line has to be rotated, using its midpoint as the rotation center, to the desired orientation before determining both parallel and perpendicular distances. The overlapping indicator d^II^(t,m) is thus the minimum displacement needed to align either the left endpoints or the right endpoints of the segments. There is an exception to this rule, if one line is within the range of the other one. In this case, the parallel shifts dII_1_ and dII_2_ are reset to zero:

(3) $${\text{d}}^{\text{II}}\left(\text{t},\text{m}\right)=\{\begin{array}{c} 0\;\text{if}\;\text{t}\subset \text{m}\;\text{or}\;\text{m}\;\subseteq \;\text{t}\\ \min\;\left({\text{dII}}_{1},{\text{dII}}_{2}\right)\;\text{otherwise} \end{array}$$

Finally, the perpendicular distance d^⊥^ (t,m) simply represents the minimum distance between two parallel lines.

#### 2.2.2. Correspondence Matrix

**Figure 5.**
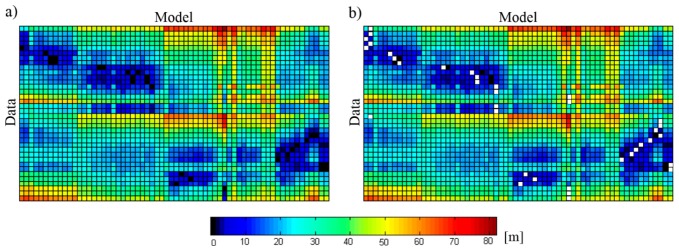
Finding the line correspondences: (**a**) Similarity matrix; (**b**) Preliminary associations S (the white squares).

Once the similarity matrix is created, one proceeds to find a pair-wise correspondence matrix (Cor) describing the relationships between two sets of lines in Data and Model. Each cell in this binary matrix filled with 0's and 1's indicates the non-existing and existing pair respectively. The correspondences are therefore found by thresholding the similarity matrix. An analysis of scores contained within the similarity matrix should be carried out to set up an appropriate threshold value δ. The process starts by linking each Data line segment with its nearest corresponding line from the Model set by means of distance d(t,m) (Figure 5).

Obviously, this task is easy to accomplish since for every line segment its most-similar feature in the other set can always be found, even if their effective distance d(t,m) is quite large or if the pairing is erroneous. Based on these preliminary one-to-one correspondences (S), an optimal threshold δ is searched by analyzing successive differences between the scores d(t,m) associated to selected pairs. The subtraction of elements is applied recursively twice.

**Figure 6.**
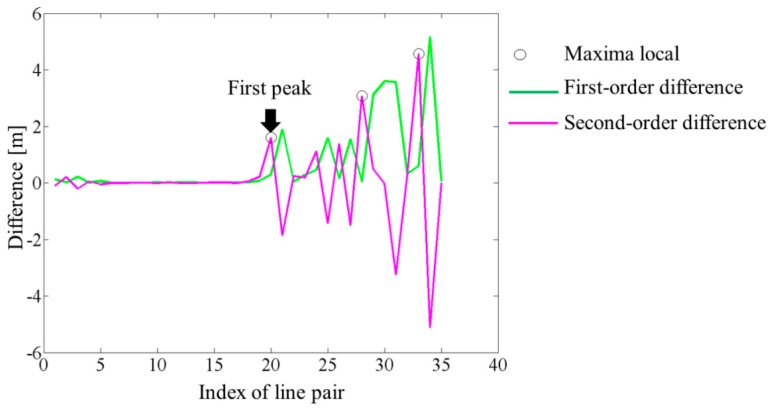
Thresholding–searching for the local maxima.

First, the differences between adjacent elements of the sequence S sorted in ascending order are calculated. This aims to create a new list of values known as the first-order difference Diff_1_ (green plot in Figure 6). The operation is carried out again, but this time on sequence Diff_1_. The result, the second-order difference (Diff_2_), is illustrated in Figure 6 (magenta plot). Next, a search for relative extrema in the input data set is used to discriminate all local maximum values in Diff_2_. Assuming an *a priori* sensibility U (minimum peak height) for the search function, the first detected peak (pks) denotes the index of the line segment pair whose score will correspond to the chosen threshold value (δ).

The above procedure is only valid for relatively large samples. Equation (4) summarizes how the threshold δ is calculated if the number of pairs N in the preliminary matching S is very low (*n* ≤ 10). For either sparse samples or when there is no local maximum (pks is an empty vector), two cases are considered: (1) Pairs within the matching S are correct while each value of Diff_1_ is insignificant (within some tolerance). The threshold δ is thus the maximum among the scores d(t,m) ordered by S; (2) The presence of gross outlier pairs in S is noted but the highest peak is smaller than U. The threshold δ is set as the median in the item S within the 2σ interval:

(4) $$\delta =\{\begin{array}{c} \max\left(\text{S}\right)\;\text{if}\;\text{N}\leq 10\;\text{and}\left|{\text{Diff}}_{1}<\text{U}\right|=\text{N}-1\\ \text{S}\left(\text{pks}\right)\;\text{if}\;\text{pks}\;\text{is}\;\text{not}\;\text{empty}\\ \tilde{\text{S}}+2\sigma \;\text{otherwise} \end{array}$$

**Figure 7.**
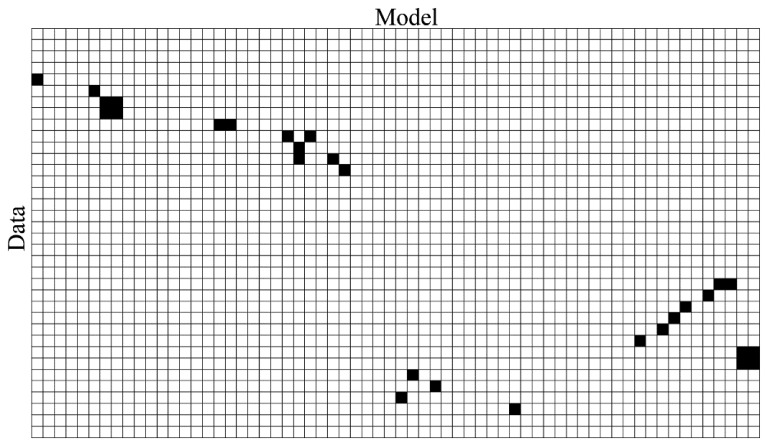
Binary correspondence matrix (Line matching is marked by the black squares).

Using this threshold δ a correspondence binary matrix Cor between the finite indexed sets Data and Model (see Figure 7) is created as:

(5) $$\text{Cor}=\left\{\left(\text{i},\text{j}\right)\in {ℵ}^{2}:\forall \left({\text{t}}_{\text{i}}\in \text{Data},{\text{m}}_{\text{j}}\in \text{Model}\right),\;\text{d}\left({\text{t}}_{\text{i}}{,\text{m}}_{\text{j}}\right)\leq \delta \right\}$$

#### 2.2.3. Computing the Hausdorff Distance between Two Sets of Line Segments-Quality Indicator

For the purpose of assessment, a quality measure, which describes the convergence between two sets of conjugate lines after the registration process, is defined. The metric used is based on the Hausdorff distance, which was originally proposed to measure the proximity of two sets of points or alternatively the similarity of shapes.

Given two datasets Data and Model as introduced earlier, the classical symmetrical Hausdorff Distance (HD) is defined as [24]:

(6) HD=max[h(Data,Model),h(Model,Data)]

The two components of the Hausdorff distance h(Data,Model) and h(Model,Data), also called directed distances, may have different values but use the same method of calculation:

(7) $$\text{h}\left(\text{Data},\text{Model}\right)=\underset{\text{t}\in \text{Data}}{\max}\underset{\text{m}\in \text{Model}}{\min}\text{d}\left(\text{t},\text{m}\right)$$

where d(t,m) denotes some underlying metric, most often the Euclidean distance. In this context, the one-sided Hausdorff distance h(Data,Model) is the maximum distance of a set Data to the nearest point in the set Model. To find the Hausdorff distance (HD), the computations must be applied reciprocally *i.e.*, from Model to Data. This classic HD measure is sensitive to noise and occlusions. Benhabiles *et al.* [25] proved the usefulness of a slightly different definition, the Modified Hausdorff Distance (MHD) [24]. The directed distance of the MHD is defined as:

(8) $${\text{h}}_{\text{MHD}}\left(\text{Data},\text{Model}\right)=\frac{1}{\left|\text{X}\right|}\sum_{\text{t}\in \text{X}}\text{d}\left(\text{t},\text{Model}\right)$$

By taking the average of the single point distances, this version decreases the impact of outliers, making it more robust than the classic HD.

The above definitions of distance are based on points and may become quite ineffectual to measure the mutual proximity of line segments. This is because they use only spatial information and neglect orientation information. On this basis, a modification of the MHD distance proposed by [23] will be employed, although in our case the computation will be carried out solely for corresponding line features. The Oriented Line Hausdorff Distance (OLHD) is defined by replacing the distance concept of the conventional HD as follows:

(9) $$\text{OLHD}\left(\text{Data},\text{Model}\right)=\frac{1}{\sum_{{\text{m}}_{\text{j}}\in \text{Model}}{\text{L}}_{{\text{m}}_{\text{j}}}}\sum_{\left(\text{i},\text{j}\right)\in \text{Cor}}{\text{L}}_{{\text{m}}_{\text{j}}}\text{d}\left({\text{t}}_{\text{i}},{\text{d}}_{\text{j}}\right)$$

where L_mj_ are the lengths of the line segments in the Model set, ordered by the correspondence matrix Cor. Since longer line segments are more reliable, the dissimilarity value d(t,m) obtained for a longer line should contribute more significantly to the symmetric LHD, our chosen measure of quality. Its value will express the degree of mismatch between two sets of registered lines. The definition of LHD from OLHD remains the same as in Equation (6) and the distance between any pair of line segments d(t,m) is calculated according to Equation (1).

### 2.3. Coarse-to-Fine Registration

A coarse-to-fine point cloud registration method based on the optimization algorithm FMII_2_ is developed in this paper to achieve robust alignment using extracted line segments. An initial search for a 3D rotation matrix (R) and translation vector (T) is applied at the beginning to improve matching accuracy. This coarse registration process is carried out in three steps: (1) Creating preliminary line matching A_ini_ between Data and Model sets; (2) Applying the RANSAC algorithm to the candidate correspondences A_ini_ to select three inlier pairs for input into the FMII_2_ algorithm (this step is mandatory because several pairs obtained previously may be incorrect); (3) Estimating the transformation parameters. Following a rough alignment of the line segments, the final transformation parameters T = (R̂,T̂) are calculated for the newly matched line segments A_f_, and used to refine the overall transformation (fine alignment).

#### 2.3.1. Solving for the Transformation Parameters

Let us first define two sets of conjugate line segments in point set Data as X and in point set Model (reference) as M which consist of N pairs. A common approach is to represent every line segment by its midpoint, noted respectively by x_i_ and a_i_, and the normalized direction vector w_i_ and v_i_:

(10) $${\text{X}}_{\text{i}}=\left({\text{x}}_{\text{i}},{\text{w}}_{\text{i}}\right)\;\text{and}\;{\text{M}}_{\text{i}}=\left({\text{a}}_{\text{i}}{,\text{v}}_{\text{i}}{,\text{L}}_{\text{i}}\right)$$

In addition, each reference line segment is described by its length L_i_. The robust optimization algorithm proposed in [17] minimizes the distance measure:

(11) $$\text{D}\left(\text{M},\text{TX}\right)=\sum_{\text{i}=1}^{\text{N}}\left[{\text{L}}_{\text{i}}\;{\left\|{\text{a}}_{\text{i}}-\text{T}-\text{R}\left({\text{x}}_{\text{i}}+{\text{s}}_{\text{i}}{\text{w}}_{\text{i}}\right)\right\|}^{2}+{\text{L}}_{\text{i}}^{3}\left(1-{\text{v}}_{\text{i}}^{\prime }\;{\text{Rw}}_{\text{i}}\right)/6\right]$$

**Figure 8.**
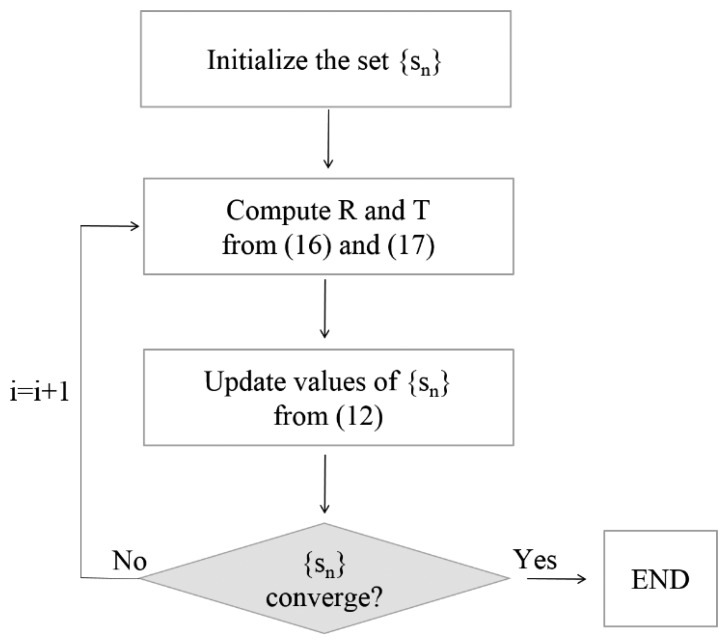
Iterative algorithm for computing the optimal transformation parameters from corresponding lines.

The cost function is defined as a sum of squared Euclidean distances of corresponding points. In our notation vectors are column matrices, ‖v_i_‖ symbolizes the length of the vector v_i_, and the prime symbol denotes a matrix transpose. For conjugate line segments, to calculate the distance D(M,TX), the corresponding points on line segments X_i_ and M_i_ must be found. This is indicated by the shift parameter s_i_: the point x_i_ + s_i_w_i_ on X_i_ denotes the point corresponding to a_i_ ϵ M_i_. The best alignment between Data and Model is obtained by minimizing D(M,TX) over all possible transformations T and {s_i_}. This nonlinear minimization problem is solved by an iterative solution as explained in Figure 8.

Thus, the six parameters of rigid transformation and the set of N shift parameters {s_i_} must be determined. Starting from the approximate values of {s_i_} set as zero, the algorithm converges when all {s_i_} cease to vary significantly with respect to the previous step. For every loop iteration, the values of the shift parameters are successively updated as:

(12) $${\text{s}}_{\text{i}}={\left({\text{a}}_{\text{i}}-\text{T}-{\text{Rx}}_{\text{i}}\right)}^{\prime }{\text{Rv}}_{\text{i}}$$

For a given set of {s_i_} the 3 × 3 cross-covariance matrix C_X,M_ is computed as:

(13) $${\text{C}}_{\text{X},\text{M}}=\sum_{\text{i}=1}^{\text{N}}\left[{\text{L}}_{\text{i}}\left({\text{a}}_{\text{i}}-\bar{\text{a}}\right){\left({\text{x}}_{\text{i}}+{\text{s}}_{\text{i}}{\text{w}}_{\text{i}}-\bar{\text{x}}\right)}^{\prime }+{\text{L}}_{\text{i}}^{3}{\text{v}}_{\text{i}}{\text{w}}_{\text{i}}^{\prime }/12\right]$$

where the centers of sets X and M are:

(14) $$\bar{\text{a}}=\frac{1}{\text{p}}\sum_{\text{i}=1}^{\text{N}}{\text{L}}_{\text{i}}{\text{a}}_{\text{i}}\bar{\text{x}}=\frac{1}{\text{p}}\sum_{\text{i}=1}^{\text{N}}{\text{L}}_{\text{i}}\left({\text{x}}_{\text{i}}+{\text{s}}_{\text{i}}{\text{w}}_{\text{i}}\right)\;\text{and}\;\text{P}=\sum_{\text{i}=1}^{\text{N}}{\text{L}}_{\text{i}}$$

The rotation matrix is found in closed-form using quaternion representation as presented in [26]. Working with the unit quaternion notation, a symmetric matrix Q_4x4_ is defined as follows:

(15) $$\begin{array}{cc} {\text{Q}}_{11}={\text{C}}_{11}+{\text{C}}_{22}+{\text{C}}_{33} & {\text{Q}}_{12}={\text{Q}}_{21}={\text{C}}_{23}-{\text{C}}_{32}\\ {\text{Q}}_{13}={\text{Q}}_{31}={\text{C}}_{31}-{\text{C}}_{13} & {\text{Q}}_{14}={\text{Q}}_{41}={\text{C}}_{12}-{\text{C}}_{21}\\ {\text{Q}}_{22}={\text{C}}_{11}-{\text{C}}_{22}-{\text{C}}_{33} & {\text{Q}}_{23}={\text{Q}}_{32}={\text{C}}_{12}+{\text{C}}_{21}\\ {\text{Q}}_{24}={\text{Q}}_{42}={\text{C}}_{31}+{\text{C}}_{13} & {\text{Q}}_{33}={\text{C}}_{22}-{\text{C}}_{33}-{\text{C}}_{11}\\ {\text{Q}}_{34}={\text{Q}}_{43}={\text{C}}_{23}+{\text{C}}_{32} & {\text{Q}}_{44}={\text{C}}_{33}-{\text{C}}_{11}-{\text{C}}_{22} \end{array}$$

The solution, namely the quaternion q = (λ_0_, λ_1_, λ_2_, λ_3_), is the eigenvector of the matrix Q associated with the largest positive eigenvalue. Finally, the orthonormal rotation matrix R from the components of the unit quaternion q is computed as:

(16) $$\text{R}=\left[\begin{array}{ccc} \lambda_{0}^{2}+\lambda_{1}^{2}-\lambda_{2}^{2}-\lambda_{3}^{2} & 2\left(\lambda_{1}\lambda_{2}-\lambda_{0}\lambda_{3}\right) & 2\left(\lambda_{1}\lambda_{3}+\lambda_{0}\lambda_{2}\right)\\ 2\left(\lambda_{1}\lambda_{2}+\lambda_{0}\lambda_{3}\right) & \lambda_{0}^{2}-\lambda_{1}^{2}+\lambda_{2}^{2}-\lambda_{3}^{2} & 2\left(\lambda_{2}\lambda_{3}-\lambda_{0}\lambda_{1}\right)\\ 2\left(\lambda_{1}\lambda_{3}-\lambda_{0}\lambda_{2}\right) & 2\left(\lambda_{2}\lambda_{3}+\lambda_{0}\lambda_{1}\right) & \lambda_{0}^{2}-\lambda_{1}^{2}-\lambda_{2}^{2}+\lambda_{3}^{2} \end{array}\right]$$

Once the rotation matrix R is calculated, the translation vector T can be computed as:

(17) $$\text{T}=\bar{a}-\text{R}\bar{x}$$

#### 2.3.2. RANSAC-Assisted Coarse Alignment

The coarse registration returns the parameters of a transformation used to achieve mutual proximity between two sets of lines. This ensures that our correspondence-finding algorithm works properly. Due to the near-certain presence of erroneous pairs in the preliminary matching A_ini_, none of optimization methods can be applied directly. Referring to [7], only K = 3 pairs of matched lines from A_ini_ should be chosen to compute an initial transformation via the robust algorithm FMII_2_. The RANSAC algorithm randomly selects K data items, which are used to estimate the parameters. The number of iterations changes dynamically as a function of the amount of inliers N_inlier_ [27]:

(18) $${\text{N}}_{\text{iter}}=\frac{\log\left(ɛ\right)}{\log\left(1-\text{q}\right)}$$

(19) $$\text{q}\approx {\left(\frac{{\text{N}}_{\text{inlier}}}{{\text{N}}_{\text{A}}}\right)}^{\text{K}}$$

where N_A_ denotes the number of pairs in the preliminary matching A_ini_, K is the number of pairs that are picked, and ε is a probability called the alarm rate (ε = 1e-6 in our case).

Once the K pairs are chosen, the estimated parameters T are validated by transforming all line segments in the Data set and then check the quality measure LHD between previously matched lines in A_ini_. Among all transformations T, the one with the “best” score is selected. The goal is to determine the value of γ that bounds, within a given probability, the error generated by a true inlier corrupted by Gaussian noise. Thus, the value γ needs to be found such that:

(20) $$\left\{\left(\text{i},\text{j}\right)\in {\text{A}}_{\text{ini}}:\forall \left({\text{t}}_{\text{i}}\in \text{X},{\text{m}}_{\text{j}}\in \text{M}\right),\text{d}\left({\text{t}}_{\text{i}},{\text{m}}_{\text{j}}\right)\leq \gamma \right\}={\text{N}}_{\text{inlier}}$$

**Pseudo-Code : Line-Based Registration, Given Two Line Sets *X_Test_* and *X_Model_* Estimate the Optimal Transformation Parameters**
Input:Coordinates of lines endpoints; σ : noise standard deviation; K: minimal sample size1Initialize : *N_I_Update_* = 0; *N_iter_* = 100; *i* = 1; ε = *1e −* 6; *k;* δ = 5.8σ2*A_ini_* ← Create preliminary ono-one correspondence between *X_Test_* and *X_Model_*3**Repeat**4*Sample* ← Select randomly *K best* data items from *A_ini_*5*R_i_*, *T_t_* ← Compute the rigid-body transformation parameters via *FMII_2_* from *Sample*6Transforme *X_Test_* and X*_Model_* : X*_Text_*___*_Update_* = *X_Test_* · *Ri* + *T_i_*7*d_i_* ← Compute all distances between matched line segments within *A_ini_* (Equation (1))8*N_I_*←(*d_i_* ≤ δ)9 **if**
*N_I_* ≥ *N_I_Update_*
**do**10  *R_ini_*← *R_i_*11  *T_ini_*←*T_i_*12  *N_I_Update_ = N_I_*13 **end if**14Update number of iteration *N_iter_ : q* ← (*N_I_*/*N*)^*k*^ ; *N_iter_* ← *log*(ε)/log(1 − *q*)15Increment : *i* ← *i* + 116**Until**
*i* ≤ *N_iter_*17*A_f_* ← Find matching between *X_Test_Upadte_* and *X_Modle_*18*R̂, T̂* ← Compute the rigid-body transformation parameters via *FMII*_2_ from *A_f_*19*R* = *R_ini_* · *R̂*20*T* = *R̂*·*T_ini_* + *T̂*21*Cor* ← *A_f_*22**Return** R T Cor


**Figure 9.**
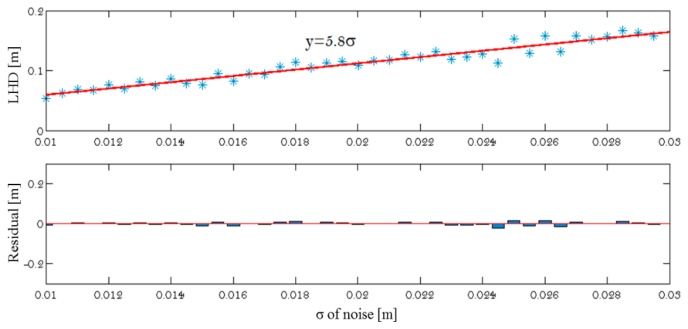
LHD between two sets of lines *versus* noise level.

In other words, the best sample is the one for which the number of line pairs from A_ini_—which after registration possess a score d(t,m) within the given tolerance γ–is the greatest. This threshold γ is set as a function of the predicted standard deviation (σ) for Gaussian noise affecting the localization of extracted line segments. The impact of noise levels on the distance has been tested by analyzing the LHD value between two datasets, the first one containing noise-free 3D line segments, the other containing the same elements corrupted by noise. Specifically, Gaussian noise with mean zero and standard deviation σ is added to the coordinates of each line segment endpoint. The relationships between the noise level and the computed distance are shown in Figure 9. The σ is directly linearly proportional to OLHD (OLHD ∝ σ) as:

(21) γ=5.8σ±0.17

Thus, if the Hausdorff distance between two sets of lines is known, the standard deviation of noise σ associated to endpoints of segments can be estimated and *vice versa*. A pseudo-code of our Matlab implementation is presented below in Procedure Line-based Registration.

## 3. Datasets and Evaluation Metrics

Two kinds of line datasets are exploited in order to assess the performance and efficiency of proposed RLMR framework.

### 3.1. Synthetic Data

**Figure 10.**
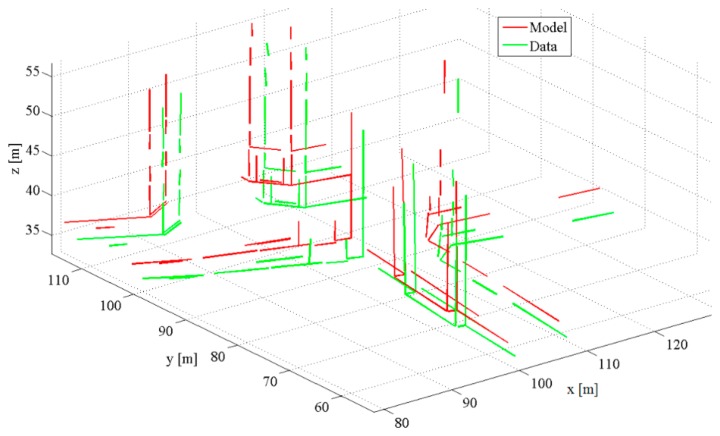
Synthetic data example.

The synthetic data consists of two datasets, each of which contains 64 linear features (Figure 10). The Data set is obtained by transforming the Model set using a rigid motion, setting the rotation (R) as Euler angles (1.0°; −1.0°; 1.0°) and the translation (T) to (−1.0 m; 0.5 m; 1.0 m).

Then, an additive white Gaussian noise with zero mean and isotropic standard deviation σ is added to the 3D coordinates of endpoints of the Model set. The noise level is successively increased from σ = 0.00 m to σ = 0.05 m with a step interval of 0.001 m. In this way, 50 noisy Data sets from a given Model set are generated. The computed Hausdorff distance (LHD) between corresponding Data and Model line segments equals to 2.892 m.

### 3.2. Real Data

The real dataset has been acquired over a dense urban area characterized by narrow (mostly one-way) streets and tall buildings where the sky visibility was limited. The data consists of one point cloud collected by a stationary Leica ScanStation C10, and three mobile scans gathered by the Stereopolis II Mobile Laser Scanning System (MLS) with the configuration described in [28].

### 3.3. Evaluation

The estimation error is given in two parts: rotation and translation. The quality of estimates (R̂) and (T̂) is thus expressed by e_R_ and e_T_ errors [15] defined by:

(22) $${\text{e}}_{\text{R}}=100\%\cdot \left\|\text{r}-\hat{\text{r}}\right\|/\left\|r\right\|$$

(23) $${\text{e}}_{\text{T}}=100\%\cdot \left\|\text{T}-\hat{\text{T}}\right\|/\left\|T\right\|$$

A vector of discrepancies is created for both rotation angles and translations. It is populated by computing the absolute differences between the estimated transformation parameters (r̂), (T̂) and the real parameters *r*,*T*. Formula (22) is valid for the axis-angle representation, which is obtained from a 3 × 3 rotation matrix (R) as explained now. The axis-angle representation parameterizes the rotation by a unit vector *r* = (e_x_,e_y_,e_z_) indicating the rotation axis and an angle θ describing the magnitude of the rotation around this axis as follows:

(24) $$\text{r}=\frac{1}{2\sin\theta }\left(\begin{array}{c} {\text{R}}_{32}-{\text{R}}_{23}\\ {\text{R}}_{13}-{\text{R}}_{31}\\ {\text{R}}_{21}-{\text{R}}_{12} \end{array}\right)$$

(25) $$\theta =\arccos\left(\frac{\text{tr}(\text{R})-1}{2}\right)$$

In turn, the performance of the proposed correspondence-finding algorithm is validated against the ground-truth. Sensitivity, specificity and accuracy are defined in terms of TP, TN, FN and FP (*cf.* Section 4.1) and used to interpret the results.

(26) $$\begin{array}{lll} \text{Sensitivity}=\frac{\text{TP}}{\left(\text{TP}+\text{FN}\right)} & & \text{Specificity}=\frac{\text{TN}}{\left(\text{TN}+\text{FP}\right)}\\ & \text{Accuracy}=\frac{\left(\text{TN}+\text{TP}\right)}{\left(\text{TN}+\text{TP}+\text{FN}+\text{FP}\right)} & \end{array}$$

## 4. Results

In the following section the efficiency and robustness of the RLMR procedure is assessed and compared to other state-of-the-art approaches, *i.e.*, EIGEN [15] and ICL (ICP form) [10]. Both synthetic and real data will be used, as well as the evaluation metrics described earlier.

### 4.1. Assessment of Estimated Transformation Parameters with Synthetic Data

First, the transformation parameters are independently computed from the synthetic data using the three algorithms mentioned above. Then, our pair-wise registration procedure RLMR, which simultaneously allows an explicit line matching, is compared to the ICL (ICP form) and EIGEN approaches for which the prior corresponding lines are known and do not have to be determined. This leads to the best possible outcome since the registration process is not perturbed by erroneous line pairings.

**Figure 11.**
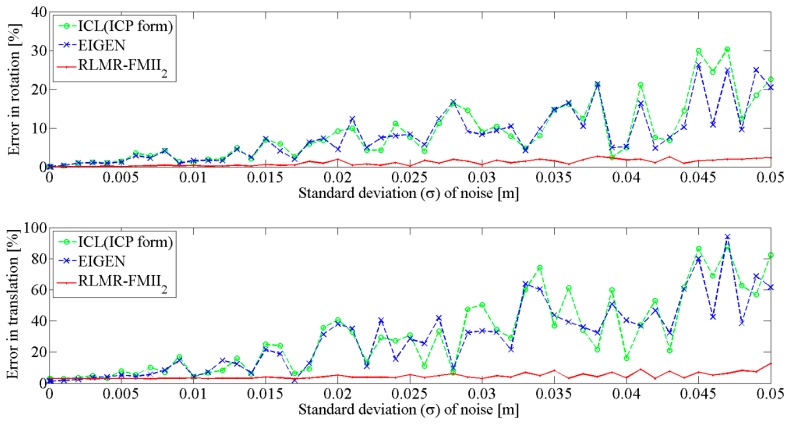
Comparison of the RLMR procedure with the state-of-the-art approaches. Error in rotation and translation.

The quality of the estimated transformation parameters, obtained from the ICL (ICP form) and EIGEN approaches, is quite similar, irrespective of noise level, as shown in Figure 11. This is logical, since both methods are analogous from an algorithmic point of view. Their main differences are the representation of line segments and rotations, as well as the way of finding optimal translation between corresponding features. However, both use an analytical method (SVD) to estimate the rotation, which is obtained from line orientations only. The precision of rotation estimation is better than 30% of full scale. The exact error value is highly dependent on the noise level. For a noise standard deviation (σ) not exceeding 0.02 m, the value is less than 10% full scale, and otherwise it fluctuates randomly. It should be noted that the error in translation e_T_ is dominant.

In contrast, the RLMR procedure gives much smaller errors than EIGEN and ICL (ICP form) methods. The results are accurate, despite the presence of an extra factor affecting the quality of the estimate, namely the matching process, which may generate some erroneous line pairings. The error in rotation e_R_ is stable regardless of the noise level. In fact, its value is less than 0.5% for σ ≤ 0.015 m, else it ranges from 0.5% to 2.8%. Finally, the error in translation e_T_ is greater than e_R_ and remains within the interval of 2.9% to 12.7%.

**Figure 12.**
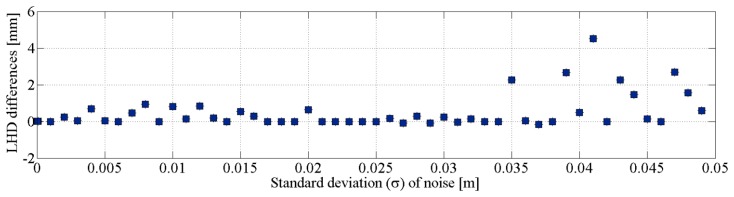
Comparison: RLMR procedure *versus* FMII_2_ under known correspondences.

In order to assess the final quality of matching (A_f_) obtained from our correspondence-finding algorithm, the results are compared with the ground-truth. First, the transformation parameters recovered by the FMII_2_ using known line associations are applied. Then, the correctness of the RLMR registration is assessed at each level of noise. The differences in LHD between the two methods are shown in Figure 12. The values are small and do not exceed 0.005 m, which confirms the robustness of our procedure and its ability to correctly identify conjugate line segments.

**Figure 13.**
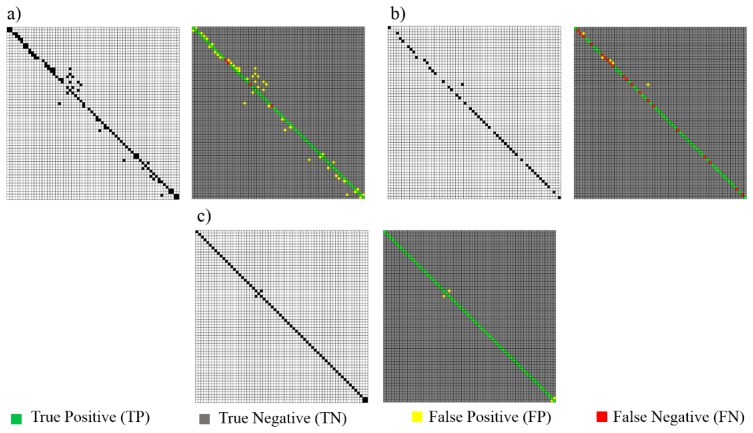
Matching accuracy: (**a**) Approximate matching A_ini_ (one-to-many relation permitted); (**b**) Final approximate matching A_ini_ (one-to-one relation at most); (**c**) Explicit matching A_f_ (without any constraints).

Afterwards, a confusion matrix (an error matrix) is created to visualize the number of pairs which are: (1) correctly matched (True Positive); (2) incorrectly identified (False Positive); (3) correctly rejected (True Negative); (4) wrongly rejected (False Negative), during both the approximate matching A_ini_ and the explicit matching A_f_. A sample result for line segments artificially corrupted by noise with σ = 0.04 m is illustrated in Figure 13.

A significant improvement in the quality of correspondence identification is observed. The assumption of lines being initially matched in at most one-to-one fashion appears to be necessary. Thus, the generation of a large amount of false positive results (FP) can be avoided at the expense of increasing the false negative (FN) detections. Seeing as the RANSAC algorithm is time-consuming in practice, this step reduces the number of iterations needed to find an optimal triad of pairs for coarse registration. Also, by decreasing the mutual distance between two datasets, the proposed correspondence-finding algorithm can ensure an exact matching.

Summarizing, the FMII_2_ optimization algorithm is preferable, especially when the linear features have significant associated uncertainty. Whenever the line correspondences are unknown, the RLMR procedure ensures a precise matching. The EIGEN and ICL (ICP form) methods cannot be applied directly due to the potentially lack of accuracy of the line localization (caused by a high noise level).

### 4.2. Assessment with Real Data

Experiments were also conducted using real point cloud (see Section 3.2). Within this data, only long (more than 2.0 m in length) and well-defined linear features were extracted via the edge-detection method described previously. The number of selected lines for subsequent tests is given in Table 1.

Based on these data, all possible permutations of {F,S_1_,S_2_,S_3_} are established. Then, the RLMR framework is used to identify the conjugate line segments, and solve for the transformation parameters.

A summary of this assessment is provided in Table 2.

The sensitivity (Equation (26)), which measures the proportion of line pairs correctly matched (TP), has an average value of 97.2%, indicating that the number of FN (the rate of non-detected existing pairs) is quite low. The specificity shows how good the matching algorithm is at identifying and rejecting non-existing pairs (TN). In our case there is on average a 99.7% chance that the candidate pair will be correctly classified as negative. The accuracy is also quite high, with none of the values lower than 99.5%. Accuracy describes the rate of the correspondence-finding algorithm obtaining true results, either TP or TN. Consequently, the proposed procedure provides an efficient and robust way to match two line sets.

The verification of convergence between registered sets of linear features is performed using the LHD. The final distance values after both rough and fine alignments are provided in Table 3, which depicts a progressive reduction of the spatial spacing between the processed datasets. Besides, the estimated transformation parameters (the rotations ω, φ, κ and translations T_x_, T_y,_ T_z_ applied respectively to the X-axis, Y-axis, and Z-axis), as well as the CPU processing time needed by our Matlab implementation of the RLMR approach (the edge-detection method processing time is omitted) are included in Table 3.

**Figure 14.**
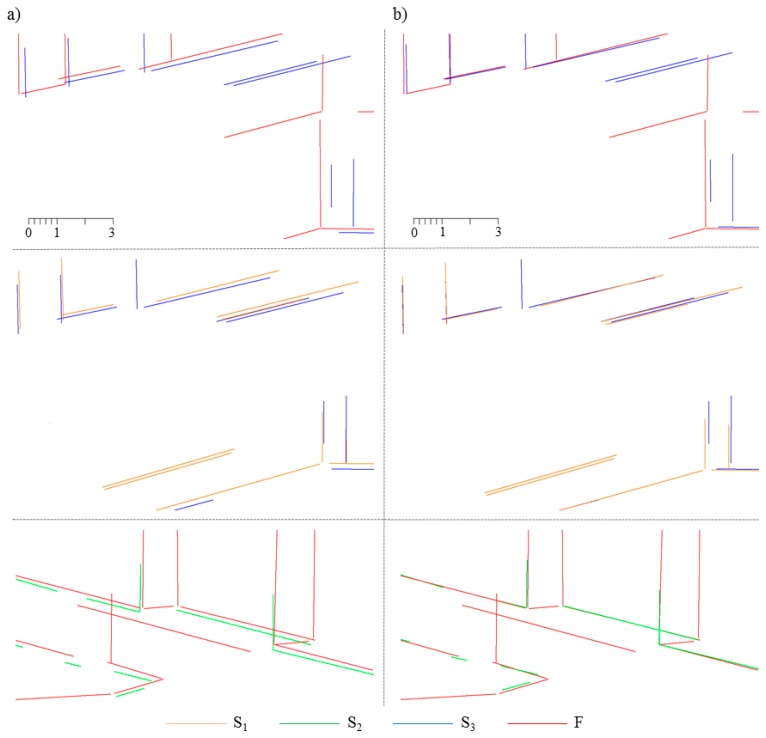
Part of line sets: (**a**) Before registration; (**b**) After registration.

To investigate the quality of the estimated transformation parameters, the registration results for a small fragment of the aligned line sets are presented in Figure 14, with different colors indicating different datasets.

## 5. Conclusions

Three main contributions of this paper are highlighted. First, a discussion of several approaches to linear feature-based registration. Second, a new correspondence-finding algorithm to match conjugate line segments, accompanied by a quality indicator which allows measuring the proximity of two line sets. Finally, the RLMR framework for performing automated pair-wise registration of low-density point clouds.

The linear feature extraction approach considers only crease edges. The proposed RLMR procedure consists of two steps: coarse and fine alignments. The coarse registration reduces the spacing between two datasets so as to improve the results of the subsequent matching process. In order to obtain a good initial transformation guess only a triplet of matched line pairs is considered. For the purpose of rejection of outlier pairs, the well-known RANSAC algorithm is used. Given two roughly registered datasets, their alignment is refined through the FMII_2,_ which employs the full set of matched lines. A novel solution for the problem of searching for conjugate matching lines was also developed. The proposed correspondence-finding algorithm exploits the positions and orientations of linear features to deduce their similarity and then pick the “same” lines within two sets. This approach may also be applied to match two sets of 2D line segments.

The application of the RLMR procedure on experimental datasets yields reliably satisfying transformation results. This framework is robust to noise and efficient if one seeks to register two initially pre-aligned point clouds at the same scale. The line extraction errors that generally comprise of laser measurement noise, occlusions, performance of the prior plane-based segmentation (over-segmentation and under-segmentation errors), do not affect the registration outcome significantly. It is due to the matching algorithm that provides a correct pairing, even if there are several linear features in one set corresponding to only one element in the other set. The quality of matching obtained in comparison tests on both real and synthetic datasets is very encouraging, provided that assumed proximity of datasets holds. This assumption is met through the coarse registration, which can considerably minimize the line matching errors as demonstrated in the result section. Thus, the rate of the proposed correspondence-finding algorithm obtaining true results is quite high, with an average value of 99.7%. Future works will focus on further evaluation of the RLMR including a comparison with other plane and point-based registration algorithms.

## Figures and Tables

**Table 1.** The number of extracted linear features from the acquired point clouds.

**Point Cloud ID**	**Number of Extracted Features**
F	64
S_1_	36
S_2_	28
S_3_	41

**Table 2.** Performance of the correspondence-finding algorithm.

**Scans ID**	**Sensitivity**	**Specificity**	**Accuracy**
S_1_-S_2_	100	99.8	99.8
S_1_-S_3_	96.6	99.7	99.6
S_2_-S_3_	100	99.8	99.8
S_1_-F	95.2	99.6	99.5
S_2_-F	95.5	99.8	99.7
S_3_-F	95.8	99.7	99.6

**Table 3.** Estimated transformation parameters for pair-wise registration by RLMR.

	**Scans ID**	**S_1_-S_2_**	**S_1_-S_3_**	**S_2_-S_3_**	**S_1_-F**	**S_2_-F**	**S_3_-F**
	Departing	0.518	0.297	0.560	0.671	1.257	0.657
LHD (m)	Coarse Registration	0.364	0.233	0.481	0.266	0.451	0.320
	Fine Registration	0.304	0.213	0.377	0.189	0.220	0.140
	ω (°)	0.35196	−0.15530	0.68971	0.30821	−0.00763	0.03841
	φ (°)	−0.12854	−0.00760	−0.19781	−0.17301	−0.14627	−0.08465
	κ (°)	−0.43047	−0.21210	0.28428	−0.01031	0.09351	0.18708
	T (m)	0.950	−0.205	1.172	−0.646	−0.331	−0.205
	T_y_ (m)	−0.833	0.108	−1.048	0.014	−0.525	−0.461
	T_z_ (m)	0.189	0.177	−0.708	−0.494	−0.285	−0.507
	* CPU Time (s)	18.1	10.4	50.4	7.8	183.6	16.4

* Central Processing Unit with Intel Core x2 T7600 2.33GHz/4 Go/Win 7 64bits.
